# Vaccination readiness and political party preference in Germany: Trust, collective responsibility, and the populist radical right

**DOI:** 10.1371/journal.pone.0328045

**Published:** 2025-07-14

**Authors:** Kathleen D. Magnus, Niklas Dammann, Daniel Lüdecke

**Affiliations:** Institute of Medical Sociology, University Medical Center Hamburg-Eppendorf, Hamburg, Germany.; Yeditepe University, TÜRKIYE

## Abstract

Numerous studies in Western countries have linked vaccine hesitancy to populist political leanings. This study focused on Germany, where there has been considerable debate as to whether this hesitancy is common across the political spectrum, fueled equally by right and left populist extremes, or driven primarily by the populist radical right. The aim of this study was to determine whether and to what extent two specific aspects of vaccine readiness -- trust and collective responsibility -- correlate with political party preferences in Germany. Evidence from a large-scale survey of German citizens (n = 2,191) showed that even after adjusting for gender, age, and education level, several indicators of vaccine hesitancy were most pronounced among supporters of Germany’s far-right populist party, the Alternative for Germany. Though these voters expressed a degree of vaccine readiness on some of the measures, they deviated significantly from all other voting groups in the direction of vaccine hesitancy on every measure. Green party voters expressed the strongest vaccine readiness, with the other major parties following close behind. The belief that the medical establishment profited excessively during the pandemic was prevalent across all parties. Taken together, these results suggest that political affiliations may play a sizeable role in views about vaccines. Results also affirm the importance that public trust and collective responsibility have for the realization of public health goals.

## Introduction

Vaccine hesitancy (VacHes) – the “delay in acceptance or refusal of vaccination despite availability of vaccination services” [1] – has been linked to populist political leanings before, during, and after the COVID-19 pandemic [2–4]. However, the nature and extent of these associations vary from country to country. In some places such as Germany, it remains unclear whether vaccine hesitancy stances are prevalent across the political spectrum, fueled by both right and left populist extremes, or driven primarily by the populist radical right.

One reason for the lack of clarity lies in the difficulty of defining the term “populism.” Although the term continues to be highly contested, most scholars agree that, at minimum, populism refers to the belief that “the will of the people” is threatened by a “corrupt elite” [5]. Typically, then, populist movements seek to divide society and undermine trust in ruling governments and scientific experts. Yet this anti-elitist, “thin” definition reveals little about actual populist movements, which tend to attach to more substantial ideologies across the political spectrum [5]. Although some researchers have discussed the substantial ideological differences among various populist movements [5–7], many theorists of populism have failed to consider these differences, even to the point of conflating starkly opposed orientations (i.e., left and right forms of populism).

To some extent, this lack of specification has been replicated in the literature on vaccination compliance and hesitancy [3,8]. For example, Kennedy’s well-known study, which analyzed data from 14 Western European countries, found a highly significant positive association between populist party voters and doubts about vaccine effectiveness and importance [2]. This pre-COVID-19 study, which included both left and right populist parties, established a significant link between the populist distrust of elites and vaccination hesitancy; however, it did not seek to determine whether doubts about vaccines are more prevalent on the left or right side of the political spectrum.

European based studies that have distinguished between left and right populist orientations have drawn various conclusions regarding vaccination hesitancy. One study based on pre-pandemic data documented considerable doubt among Europeans as to the safety of vaccinations, noting that this doubt was especially strong among ideological extremists “on both ends of the spectrum” [9]. By contrast, another study based on data gathered during the same period concluded that neither general populist party support, nor far-right populist party support could predict vaccine hesitancy [10]. Further studies, including two that focused on the COVID-19 vaccinations, found only right-wing political orientations to have a negative impact on vaccine intention and behavior [4,11].

The case is similar with respect to Germany in particular. Here too research has suggested a strong association between populism and vaccine hesitancy [12,13], but studies on vaccine hesitancy that distinguish among various forms of populism or various political orientations remain relatively rare. Even where political party affiliations have been taken into account, research on their role in vaccine hesitancy remains inconclusive, and considerable disagreement persists as to whether party affiliations play a significant role in vaccine views and behavior in Germany. One 2023 study found that party affiliation, political ideology, and region of residence each impacted vaccine hesitancy more significantly than gender and educational background [14], two factors shown elsewhere to be significant [15]. In the same year, however, another study found political leanings to have little relevance once other variables, such as regional differences were controlled for [16]. Other studies found differences in vaccine willingness to vary along political lines, but they diverged in their conclusions as to exactly how these divergences play out. Some observed significant vaccination hesitancy across the political spectrum [17], while others found this hesitancy only on the left and right extremes and among members of the newly formed protest parties, with these associations moderated by age, gender and region of residency [14]. In contrast to both of these positions, another set of studies found stark differences between those that adhere to left and those that adhere to right political orientations [18,19].

These mixed results suggest the need for further investigation into how political party affiliations may be related to positions and behaviors regarding vaccines in Germany. In order to clarify this issue, we assessed political party affiliation based on voting behavior in Germany’s 2021 federal election, focusing on the six main political parties in Germany. Using this metric allowed us to compare populist parties with more mainstream parties without conflating the populist radical right (*Die Alternative für Deutschland*, that is, The Alternative for Germany, known as the “AfD”) [20,21] with the populist left (often associated with *Die Linke* (The Left Party) [22].

In contrast to vaccine hesitancy, which connotes a reluctance to get vaccinated, “vaccine readiness” designates the inclination or willingness to do so. This paper focuses on two key elements of vaccine readiness: 1) trust in vaccinations, i.e., trust in science, health authorities, government elites that pertain to the development and distribution of vaccines, and (2) a sense of collective responsibility. Due to the unique circumstances surrounding the COVID-19 pandemic, we also posed two questions pertaining specifically to issues of trust surrounding the production and distribution and effectiveness of the COVID-19 vaccine.

To date, there has been considerable research on the role that trust plays in vaccine readiness, especially in light of the COVID-19 pandemic. Not surprisingly, high levels of trust in scientific experts and governmental institutions are associated with greater COVID-19 vaccine intention [19,23] and/or positive vaccine status [24], while low levels of trust in these areas have been correlated with vaccine hesitancy and refusal [3,25]. More difficult to ascertain, however, are the complex, interacting factors that build and deplete this trust. Numerous studies suggest that a lack of trust in vaccines is strongly associated with exposure to false information and conspiracy theories [26,27], the propagation and acceptance of which are typical among populists [28,29]. Yet populists are not the only actors who seek to promote distrust, nor is misinformation the sole reason for medical and government skepticism. Along with a host of other factors that contribute to vaccine hesitation [4,15,23], there is considerable evidence to suggest that specific ideologically linked political parties may harbor more vaccine skepticism than other parties [11,30,31], even if reasons for the mistrust of governments may vary within a single party [32].

Less attention has been paid to the role that a sense of collective responsibility among German citizens plays in vaccine readiness, though some researchers have acknowledged the importance of this factor [33–35]. Given that vaccines protect not only the individuals who get vaccinated, but also those who come into contact with them, it is likely that vaccine willingness is at least partly motivated by a sense of collective responsibility and linked to what has been called “prosocial behavior” [33]. Conversely, a prioritization of individual freedom has been associated with vaccine hesitancy [24,36]. Hypothesizing that levels of trust and collective responsibility are likely to be correlated with political party affiliations and voting behavior, we sought to determine the impact of each of these factors on vaccine hesitancy and to assess their relevance within Germany’s six largest political parties as of 2021. In addition, due to the unique circumstances surrounding the production and distribution of the COVID-19 vaccination, we hypothesized that additional concerns leading to vaccine hesitancy may have been operative during the pandemic.

## Methodology

### Ethical approval

This study was approved by the Local Psychological Ethics Committee of the University Medical Center Hamburg–Eppendorf (LPEK-0667). Participants consented through an online digital opt-in process conducted by the market research firm YouGov from March 1–5, 2024. Participants were informed that they could skip any question considered sensitive and that they could discontinue the survey at any time. No minors were included.

### Sampling and participants

This survey was conducted from March 1–5, 2024 by YouGov, an internet-based market research and data analytics firm based in the United Kingdom. Participants included eligible German voters aged 18 and up (n = 2,191), who were invited via email with a link to YouGov’s online omnibus survey. All results are based on a sample that was drawn at random from the population of the YouGov Online panel. However, because response rates can vary, in addition to the quota frame, a weighting factor was calculated considering age, gender, education, place of residence (rural, suburban, urban), region (federal state), voting behavior in the last federal election, and political interest in order to ensure a target distribution that is representative of the population. Education categories followed YouGov’s standard classification: “low” included those still in school, those without a degree and those possessing a general degree (*“Haupt- oder Volksschulabschluss”*); “medium” included those with a mid-level degree (*“Realschulabschluss oder gleichwertiger Abschluss”*) and “high” included those with an advanced high school degree (*“Abitur”*) and higher. Gender categories also followed YouGov’s binary delineation. In defining and weighting quotas, YouGov is guided by official population statistics (e.g., *Mikrozensus*, European Social Survey, and the *Bundeswahlleiterin*). YouGov maintains internal standards that comply with ISO 9000 and has an ISO 27001 certified information security management system.

### Development of the questionnaire

Our questionnaire drew heavily from the work of Betsch and colleagues (2018), which delineated five components of vaccine readiness [37]. We focused on the elements of vaccine readiness related to *trust* and *collective responsibility* because we hypothesized that differences in these areas would fall along party lines. Here “trust” is used in the place of the word “confidence,” which has been defined by the World Health Organization’s EURO Working Group on Vaccine Communications [38] in terms of trust, specifically as “trust in (i) the effectiveness and safety of vaccines; (ii) the system that delivers them, including the reliability and competence of the health services and health professionals and (iii) the motivations of policy-makers who decide on the needed vaccines” [1]. With regard to “collective responsibility,” we follow the definition proposed by Betsch and colleagues, namely, “the willingness to protect others by one’s own vaccination by means of herd immunity” [39]. Overall, the survey included nine items, six that drew from Betsch and colleagues and three original questions, which tested for additional aspects of trust. In each case, participants were asked to respond to a series of statements, using the following scale: 1 = disagree completely, 2 = tend to disagree, 3 = tend to agree, and 4 = agree completely. The survey was conducted in German. The original German and English versions are both available as supplementary material (Supplementary Files S1 and S2). The survey also included a question asking about the number of COVID-19 vaccinations survey participants received, though this was not part of our main analysis (See S3). The questionnaire was evaluated for content and pilot-tested by a panel of ten experts.

### Dependent and independent variables

We developed four dependent variables, each representing a component of vaccine readiness. These included two sets of composites: one for “trust in vaccinations” (Q1-Q3) and one for “collective responsibility” (Q4-Q7) as well as two single items (Q8 and Q9). Responses to Q8 and Q9, which pertained specifically to issues of trust during the COVID-19 pandemic, were not included in either composite because the circumstances surrounding the development and administration of these vaccines were unique.

The first composite representing trust in vaccinations asked participants to respond to three statements (question numbers in parentheses): ‘(1) I have full confidence in the safety of vaccinations’; ‘(2) Vaccinations are effective for the containment of infectious diseases’; and ‘(3) When it comes to vaccinations, I always trust state authorities to decide in the best interest of the public.’ This set of questions was previously validated [37]. For our data set Cronbach’s alpha was 0.853.

The second composite representing “collective responsibility” asked participants to respond to four statements: ‘(4) If everyone is vaccinated, I don’t need to get vaccinated’; ‘(5) I get vaccinated because I can also protect people with a weak immune system’; ‘(6) Whether or not to get vaccinated is a purely personal decision’; and ‘(7) Vaccination is a community measure to prevent the spread of disease.’ Questions 4, 5, and 7 were prevalidated [37]. Since we introduced a new question (Q6) into this composite set, we performed a principal component analysis with varimax rotation to assure its internal consistency. Cronbach’s alpha for this composite score was 0.687.

Question 8 represents “trust in medical institutions” and asked survey participants to consider the statement: ‘Many pharmaceutical companies, hospitals and medical doctors profited excessively from the COVID-19 pandemic.’ Question 9 asked survey participants to consider the statement: ‘In Germany vaccinations were able to contain the COVID-19 pandemic.’ The statement draws attention to the German government’s role in developing and distributing the vaccine and so serves as a measure of “trust in government.” Where necessary individual items were recoded so that in each case a higher score represents a higher level of vaccine readiness (i.e., more trust, more collective responsibility, etc.). Invalid responses (i.e., those who did not vote, voted for small parties outside the scope of our study, marked ‘I don’t know’ or did not provide an answer) were excluded from our calculations. No response rates are listed according to party voting behavior in the supplementary material (S4).

Participants were asked to identify the political party for which they voted in 2021. Based on this identification, responses were sorted into six groups indicating the six political parties which received the most votes in Germany’s 2021 Federal Election: The Left party (*Die Linke*); The Green party (*Bündnis 90/Die Grünen*); The Social Democratic Party, (*Sozialdemokratische Partei Deutschlands* known as the “SPD”); The Christian Democratic Union (*Christlich Demokratische Union* measured together with its sister party and abbreviated here as the CDU/CSU); The Free Democratic Party (*Freie Demokratische Partei* known as the FDP); and the Alternative for Germany (*Alternative für Deutschland*, known as the AfD) [40]. These six parties served as the independent variables. Participants who voted for another party, did not vote, or responded ‘I don’t know’ were excluded from the analysis.

### Data analysis

**Fig 1:**
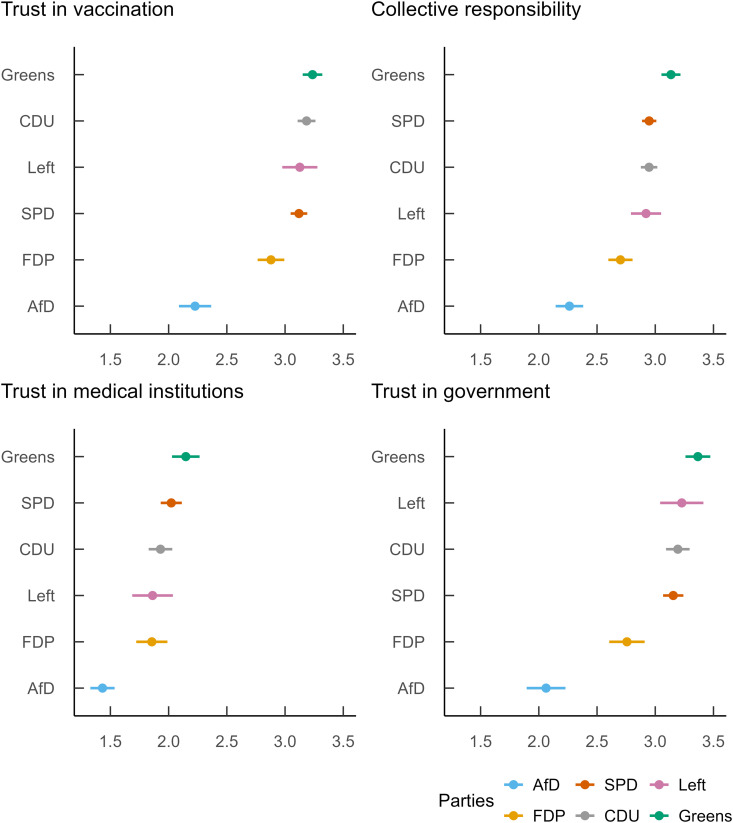
Vaccine readiness: Estimated marginal means for each outcome by party preference.

Characteristics of the sample, stratified by political party preferences, are reported as means with standard deviation for numeric and proportions for categorical predictors. Cronbach’s alpha values were calculated for the two composite scores to check the internal consistency. We performed linear regressions to measure the associations between each of the six main political parties and each of the four outcomes, controlling for age, gender, and education level. The estimated marginal means were calculated for each regression model using heteroskedasticity consistent standard errors. Point estimates and 95% confidence intervals are shown in the Fig 1. Post-hoc pairwise comparisons were used to determine differences between the individual parties. Statistically significant differences are emphasized in the tables. All analyses were weighted according to the stipulations of YouGov. All analyses were performed using R 4.4.2 [41], using packages *datawizard* and *ggeffects* [42,43].

## Results

### 1) Description of Sample

As Table 1 indicates, respondents were 51.5% male and 48.5% female. Of those surveyed, 28.5% were between 18 and 39 years of age; 33,7% were between 40 and 59; and 37.8% were 60 or older. A total of 25.8% of respondents had an education level categorized as low; 29.5% as medium, and 44.7% as high.

**Table 1:** Description of Sample.

Descriptive Statistics (weighted)							
Variable	SPD (n = 448)	CDU (n = 419)	Greens (n = 258)	AfD (n = 181)	FDP (n = 199)	Left (n = 86)	Total (n = 1605)
*Outcomes*							
Mean Trust in Vaccinations (SD)	3.13 (0.73)	3.21 (0.72)	3.27 (0.63)	2.23 (0.94)	2.90 (0.76)	3.14 (0.72)	3.04 (0.80)
Mean Collective Responsibility (SD)	2.98 (0.63)	2.98 (0.67)	3.17 (0.62)	2.29 (0.79)	2.71 (0.70)	2.93 (0.64)	2.90 (0.71)
Mean Trust in Medical Institutions (SD)	2.03 (0.88)	1.95 (0.90)	2.21 (0.88)	1.41 (0.69)	1.90 (0.85)	1.89 (0.79)	1.94 (0.88)
Mean Trust in Government (SD)	3.21 (0.84)	3.24 (0.90)	3.41 (0.76)	2.09 (1.09)	2.79 (0.99)	3.27 (0.80)	3.08 (0.97)
*Sex*							
female%	51.8	50.2	52.6	41.0	43.4	38.7	48.5
*Age Groups*							
18-39, %	25.8	26.5	36.3	15.4	36.7	38.3	28.5
40-59, %	26.8	34.1	36.2	46.4	34.7	31.0	33.7
60 + , %	47.4	39.4	27.6	38.2	28.6	30.7	37.8
*Education*							
Low, %	32.5	31.5	10.7	29.5	18.7	18.6	25.8
Mid, %	31.7	27.5	19.8	44.5	28.8	27.5	29.5
High, %	35.8	41.0	69.6	26.0	52.5	53.9	44.7

### 2) Political party averages of the four outcomes measuring vaccine readiness

Fig 1 presents the political party averages (estimated marginal means) for each of the four outcomes representing different aspects of vaccine readiness. Results show that voters from all parties except the AfD tended toward vaccine readiness on three of the four measures. On the third outcome, which measured the belief that medical and pharmaceutical professionals profited “excessively,” voters from every party signaled some vaccine hesitancy.

Results of the first outcome showed that Green party voters expressed the most trust in vaccines (3.24), followed by the CDU voters (3.18). AfD voters expressed the least trust in vaccines (2.23), with FDP showing the second lowest level (2.88). SPD, Left, and CDU voters fell in the range of 3.12–3.18.

Results of the collective responsibility composite (i.e., concern about protecting others through vaccination) produced the second outcome. These results were generally lower than the results relating to trust in vaccinations (though the AfD score here was slightly higher than in the first outcome). The estimated marginal means of AfD voters was nonetheless considerably lower (2.26) than that of all other parties. FDP party voters had the next lowest score (2.70), tending more in the direction of vaccine readiness. Again, Green party voters expressed the strongest sense of collective responsibility (3.13) with SPD, Left and CDU voters falling in the range of 2.92–2.95.

As indicated above, average scores from all parties showed considerable agreement with the notion that pharmaceutical companies, hospitals, and medical doctors profited excessively from the COVID-19 pandemic (3^rd^ outcome in Fig 1). Here, AfD and FDP voters agreed the most emphatically, signaling a lower level of vaccine readiness (reverse-coded: AfD = 1.43; FDP = 1.86), while Green voters proved to be the least suspicious of unjustified profits (reverse-coded: 2.15).

AfD voters also stood out in their rejection (2.06 average) of the notion that vaccinations served to contain the spread of the COVID-19 in Germany (4th Outcome in Fig 1). With the exception of AfD voters, responses were generally positive (2.76 or higher). Green party voters also showed the highest readiness score in this category (3.37).

### 3) Trust in vaccinations

Table 2 shows the results of the post-hoc pairwise comparisons based on the linear regressions controlling for age, gender, and education level. AfD voters deviated significantly (p < 0.001) in the negative direction from each of the other main parties (the SPD, CDU/CSU, The Greens, the FDP and the Left party). The FDP deviated negatively and significantly from all parties except the AfD, from which its significant deviation was positive. Green party voters expressed the strongest trust level, deviating positively and significantly (p < 0.001) from the FDP and AfD and to a lesser extent (p < 0.05) from the SPD.

**Table 2:** Trust in vaccinations. Results of pairwise comparisons based on linear regressions.

Ref. Party	Deviation from reference party						
		Left	FDP	AfD	Green	CDU/CSU	SPD
	**Left**	∕	−0.248*	−0.899***	0.109	0.058	−0.007
	**FDP**	0.248*	∕	−0.652***	0.357***	0.305***	0.240***
	**AfD**	0.899***	0.652***	∕	1.008***	0.957***	0.892***
	**Green**	−0.109	−0.357***	−1.008***	∕	−0.051	−0.116*
	**CDU/CSU**	−0.058	−0.305***	−0.957***	0.051	∕	−0.065
	**SPD**	0.007	−0.240***	−0.892***	0.116*	0.065	∕

These calculations were based on total responses (n = 1581) and controlled for age, gender, and education level. *p < 0.05, **p < 0.01, ***p < 0.001.

### 4) Sense of collective responsibility

Pairwise comparisons based on linear regressions controlling for age, gender, and education level determined AfD voters to have the lowest level of vaccine-related collective responsibility, a result that deviated significantly (p < 0.001) from all other parties (Table 3). FDP voters also deviated significantly in the negative direction from the Greens, CDU/CSU, SPD and to a lesser degree from the Left party. Green party voters showed the strongest sense of collective responsibility, deviating significantly (p < 0.001) in the positive direction from voters from all other parties, but to a lesser extent (p < 0.01) from Left party voters. The Left party did not differ significantly from either the SPD or the CDU.

**Table 3:** Sense of collective responsibility. Results of pairwise comparisons based on linear regressions.

Ref. Party	Deviation from reference party						
		Left	FDP	AfD	Green	CDU/CSU	SPD
	**Left**	∕	−0.220**	−0.658***	0.214**	0.026	0.027
	**FDP**	0.220**	∕	−0.438***	0.434***	0.246***	0.247***
	**AfD**	0.658***	0.438***	∕	0.872***	0.684***	0.685***
	**Green**	−0.214**	−0.434***	−0.872***	∕	−0.188***	−0.187***
	**CDU/CSU**	−0.026	−0.246***	−0.684***	0.188***	∕	0.001
	**SPD**	−0.027	−0.247***	−0.685 ***	0.187***	−0.001	∕

These calculations were based on total responses (n = 1585) and controlled for age, gender, and education level. *p < 0.05, **p < 0.01, ***p < 0.001

### 5) Variations of trust during the COVID-19 pandemic

As noted above, Question 8 asked survey participants to consider whether and to what extent they believed that pharmaceutical companies, hospitals and medical doctors profited excessively from the COVID-19 pandemic. Pairwise comparisons based on linear regressions controlling for age, gender, and education level determined the AfD’s deviation from the other parties to be significant (p < 0.001) in the negative direction (Table 4). The FDP deviated significantly (p < 0.001) from the AfD in the positive direction but also deviated considerably in the negative direction from the SPD (p < 0.01) and the Greens (p < 0.001). Left party voters fell in the range of the mainstream parties, deviating positively and significantly from the AfD (p < 0.001), but somewhat negatively from the Greens (p < 0.05).

**Table 4:** Trust in medical institutions. Results of pairwise comparisons based on linear regressions.

Ref. Party	Deviation from reference party						
		Left	FDP	AfD	Green	CDU/CSU	SPD
	**Left**	∕	−0.006	−0.430***	0.285**	0.068	0.161
	**FDP**	0.006	∕	−0.424***	0.292**	0.074	0.167*
	**AfD**	0.430***	0.424***	∕	0.715***	0.498***	0.591***
	**Green**	−0.285**	−0.292**	−0.715***	∕	−0.217**	−0.125*
	**CDU/CSU**	−0.068	−0.074	−0.498***	0.217**	∕	0.093
	**SPD**	−0.161	−0.167*	−0.591***	0.125*	−0.093	∕

Calculations based on total responses (n = 1446) and controlled for age, gender, and education level. *p < 0.05, **p < 0.01, ***p < 0.001.

Responses to Question 9, asking survey participants to consider whether and to what extent vaccinations in Germany were able to contain the COVID-19 pandemic, comprised the 4^th^ outcome (Table 5). AfD voters expressed considerably less agreement with the view that Germany was successful in containing the COVID-19 pandemic than other voters. Here too results of a pairwise comparison based on linear regressions, controlling for age, gender, and education level showed that AfD voters deviated negatively and significantly (p < 0.001) from voters of all other parties (Table 5). Although FDP voters tended to agree with the statement, their agreement was significantly weaker (p < 0.001) than that registered by all other parties except the AfD from whom their deviation was significantly positive (p < 0.001). The Greens differed significantly (but to various degrees) in the positive direction from all parties except the Left party.

**Table 5:** Trust in government. Results of pairwise comparisons based on linear regressions.

Ref. Party	Deviation from reference party						
		Left	FDP	AfD	Green	CDU/CSU	SPD
	**Left**	∕	−0.471***	−1.166***	0.138	−0.034	−0.073
	**FDP**	0.471***	∕	−0.695***	0.609***	0.437***	0.398***
	**AfD**	1.166***	0.695***	∕	1.304***	1.132***	1.092***
	**Green**	−0.138	−0.609***	−1.304***	∕	−0.172*	−0.212**
	**CDU/CSU**	0.034	−0.437***	−1.132***	0.172*	∕	−0.039
	**SPD**	0.073	−0.398***	−1.092***	0.212**	0.039	∕

These calculations were based on total responses (n = 1478) and controlled for age, gender, and education level. *p < 0.05, **p < 0.01, ***p < 0.001

Results according to political party voting for each separate item can be found in the supplementary material (S5).

## Discussion

Our study was able to determine several ways in which German political party preferences correlated with certain vaccine-related positions. Though we did not measure every aspect of vaccine readiness, we were able to show that *trust in vaccines* (along with the process by which they are developed and administered) as well as “*collective responsibility”* (understood in this context as the willingness to get vaccinated in order to protect others) could be linked to political party preferences. In addition, within the context of the COVID-19 pandemic, we identified some particular concerns related to trust which are not usually tested for, and we were able to demonstrate that these may be linked to political party preferences. Due to the complex interaction of factors beyond the scope of this study [44,45], as well as the possibility of reverse causality [46], these results should not be interpreted as establishing any causal relationships. Nonetheless, our study identified several significant patterns.

Most notably, the study indicated that, even after covariate controls, AfD voters tended toward the most vaccine-hesitant positions, deviating significantly from voters of all other political parties in the direction of vaccine hesitancy. This overall result suggests that vaccine hesitancy in Germany is a predominantly radical right populist phenomenon. It also is worth noting that although the FDP deviated significantly in the positive direction from the AfD (expressing a higher vaccine readiness) on every outcome, these voters also deviated significantly in the negative direction from the CDU/CSU, and the Left party on three of the four outcomes and from the SPD and Greens on all outcomes. In other words, FDP voters consistently had the second lowest scores for vaccine readiness.

Furthermore, our analyses showed that, even after covariate controls, Green party voters yielded the highest vaccine readiness scores. In addition, Green party voters differed significantly in the direction of vaccine readiness from other mainstream party voters on our collective responsibility measure, and they expressed considerably less concern about medical profiteering than those voting for the other parties (3^rd^ outcome). The pattern is similar with respect to the perceived success of Germany’s handling of the pandemic (4^th^ outcome), except that Green party voters did not deviate significantly from Left party voters, who also expressed a high satisfaction with Germany’s handling of the crisis.

Finally, and contrary to some studies which have suggested that vaccine hesitancy stems from populist positions on both sides of the political spectrum, our results indicate that, at least in Germany, Left party voters land solidly in the domain of mainstream with regard to vaccination. Not only did they deviate significantly from the AfD in the direction of vaccine readiness on all of the measures, but they also deviated significantly and positively from the FDP on the trust and collective responsibility scores, as well as with regard to the perceived success of Germany’s handling of the COVID-19 pandemic.

Our analysis thus highlights the extent to which AfD voters fall outside of the mainstream on issues related to vaccination. However, it would be wrong to conclude that AfD voters are extremely hesitant when it comes to vaccines. Given that AfD voters averaged above 2 on three of our four outcomes (Fig 1), it would be more accurate to conclude that this voting group expressed a *tendency* towards vaccine hesitancy. In the case of the third outcome, where they averaged below 2 (and were therefore clearly in the domain of vaccine hesitancy), they were not alone, as FDP, Left, and CDU party voters also averaged below 2 on this measure.

The results pertaining to our “trust” composite underline the importance of the public’s confidence in the safety and effectiveness of vaccines and in the decisions of public health and governmental officials. At the same time, they reveal how weaknesses in these areas may be linked to vaccine hesitancy. Through the inclusion of a question not featured in most standard vaccine-hesitancy or vaccine-readiness scales (Q8, Outcome #3), our study was able to identify an additional factor related to trust that proved relevant with respect to the COVID-19 pandemic. Specifically, our results showed that a majority of respondents across the political spectrum were concerned about the “excessive” profits made by pharmaceutical companies, hospitals, and medical doctors during the COVID-19 pandemic. With the exception of FDP voters, average scores in response to this question were the lowest for each party (ranging from 1.43 to 2.15), indicating that this is an area where trust may be the most lacking. For this reason, further studies should be conducted to determine exactly why this might be the case. Although this lack of trust may have been especially prevalent during the pandemic and in relation to the COVID-19 vaccine, low scores on this measure may indicate a broader suspicion of medical profiteering. Further studies will be needed to determine if this is the case. One possible interpretation of our results in this area may be that some individuals suspect that vaccines are promoted for profit, rather than for purely scientific reasons. Given that concerns about corruption have been shown to be highly relevant to individuals’ health decisions [47], it would not be surprising if this suspicion regarding medical profiteering proved to be a major driving force in vaccine hesitancy. Therefore, if the achievement of herd immunity through widespread vaccinations is to remain a feasible public health goal, public health officials will need to consider this concern and respond to it in their vaccine campaigns. Public suspicion of corruption might also be reduced if pharmaceutical companies, hospitals, and medical doctors were required to offer financial transparency regarding the profits that they garner from the production and distribution of vaccinations.

With respect to the issue of collective responsibility, our results were a bit more nuanced. In the case of the AfD, there was some indication that issues related to collective responsibility drove vaccine hesitancy to a lesser extent than issues related to trust. However, in general, the scores pertaining to collective responsibility were notably lower than the scores pertaining to trust (Fig 1). Given that a strong sense of collective responsibility is necessary for a society to achieve herd immunity, our results suggest that educational and governmental officials in Germany should invest more in promoting a sense of shared responsibility for the common good. Educating citizens about the ways in which their individual fates are intertwined with the health and stability of their society could help them see that individual and social goals need not conflict, but may in fact support each other. Specifically with regard to the issue of vaccination, knowledge of the protective benefits of herd immunity would allow individuals to see that even if they do not feel directly threatened by any given infectious disease, protecting others from such diseases reduces the chances of mutations that may be more threatening to everyone – themselves included. In addition, utilizing available vaccinations could reduce the need for lockdowns and other extreme measures used to control viruses, thus maintaining a higher level of economic productivity and individual freedom for all. Even though the German citizens we surveyed generally understood vaccination to be a community endeavor, they also held fast to the idea that the decision whether or not to get vaccinated is a “purely personal” one (See S5). This may be something for health officials to keep in mind since it suggests the possibility that vaccine mandates could backfire in some segments of the population. Improving health education and enacting campaigns that nudge behavior toward public health compliance may be more effective ways to overcome vaccine resistance.

Our study has both strengths and limitations. We focused on German citizens’ views of vaccines in general in order to investigate whether and to what extent vaccine-related positions may be linked to specific voting patterns. Although we included some questions referring specifically to the COVID-19 vaccine, it was beyond the scope of our study to consider whether and to what extent voting patterns might vary according to the particular vaccination and specific disease in question. Given that misinformation and skepticism surrounding the COVID-19 vaccines were particularly high during the pandemic, it is possible that individuals’ experiences with the COVID-19 vaccine(s) may have influenced their general position on vaccines. Future studies will need to consider whether, how, and to what extent this may be the case. The purpose of our study, however, was not to measure changes over time, but rather to determine the levels of vaccine hesitancy and vaccine readiness in current post-pandemic Germany.

Due to the relatively low percentage (4,9%) of votes garnered by the Left party in Germany’s 2021 election [40], this study could include only the correspondingly small sample size. Results pertaining to this party should therefore be interpreted with caution. Moreover, although the study controlled for factors such as education, age, and gender, the study did not control for factors such as income level and place of residence. It is also worth noting that the two measures we introduced, Q6 (vaccination as a purely personal decision) and Q8 (trust in pharmaceutical and medical institutions and experts) yielded relatively low averages across the political spectrum. (S5). It was also only in response to these two questions that any of the voting groups besides AfD voters expressed a tendency toward vaccine hesitancy. We therefore recommend that further studies be conducted to determine the precise extent to which these measures reflect vaccine hesitancy and/or readiness. Especially with regard to Q6, we recommend further studies, as its inclusion in our collective responsibility composite was based on a borderline Cronbach’s alpha. At the same time – and precisely because the overall results from these questions were lower than the results from other questions – we urge further consideration of these factors as likely contributors to vaccine hesitancy. This is especially important given that the issues raised in these questions – concerns for personal freedom and suspicions about corruption – play an important role in the kind of medical care people seek [47] and often feature (explicitly or implicitly) in right-wing propaganda [5,48]. These issues may well drive vaccine hesitancy to a greater extent than has been acknowledged to date.

Building on research that supports a link between the rise of vaccine hesitancy and the rise of populism, this study highlights the complexity of the connection. By showing the relationship between various political party preferences on the one hand and vaccine readiness/hesitancy on the other, our study draws attention to how elements of populism may be intricately intertwined with various political ideologies and agendas. Though the distrust of elites is common to all populist movements, our findings indicate that, at least in Germany, vaccine hesitancy is driven more by right-wing populist forces than by left wing populist movements. This does not gainsay the existence of the latter. Indeed, populist distrust may arise from a variety of sources and be directed toward a variety of targets. A prime example of this can be found in the USA, where both of the major political parties include populist subgroups, whose members tend to distrust different kinds of elites. According to a recent Gallup poll, only 69% of respondents consider vaccination of children to be “very” or “extremely important,” (down from 94% in 2001), but this reduction in vaccine readiness is strongly driven by members of the Republican party [49]. The considerable amount of vaccine hesitancy among Republicans reflects a distrust of scientific and bureaucratic elites that is less common among Democrats, who are more likely to focus on the corruption of the corporate elite. In this case, then, what drives vaccine hesitancy seems not to be populist sentiment *per se*, but rather a particular combination of populist elements and right-wing ideology.

More studies will need to be done to determine whether a similar dynamic is operative in Germany. What is clear is that vaccine hesitancy and right-wing populism are on the rise in several countries throughout Western society [50,51]. Now that vaccine hesitancy has become significant enough to permit outbreaks of preventable diseases [52], it is especially urgent that social scientists clarify the impact that radical right populist forces have on citizens’ vaccination views. Of course, a range of studies will be required to fully explain the complex, interacting relationships among political party ideologies, populist views, and vaccination positions. We hope our study serves as an impetus for future research in these areas.

## Supporting information

S1. Table Survey Results Vaccine readiness (in German). (PDF)

S2. Table Survey Results Vaccine readiness (in English). (PDF)

S3. Table COVID-19 Vaccinations received, listed by voting behavior in the Bundestag elections 2021. (PDF)

S4. Table Number of ‘no responses,’ listed by voting behavior in the Bundestag elections 2021. (PDF)

S5. Table with explication. Nine item averages according to political party.
(DOCX)
