# Supplementary material for: Vaccination readiness and political party preference in Germany: Trust, collective responsibility, and the populist radical right
Source: PLoS One. 2025 Jul 14;20(7):e0328045. doi: 10.1371/journal.pone.0328045 (PMC12258577; doi:10.1371/journal.pone.0328045)
Supplement: S1. Table — (PDF) [file pone.0328045.s001.pdf]

S1 Table. Survey Results: Vaccine readiness (in German)

Anzahl Teilnehmer	Total	SPD	CDU/CSU	Bündnis 90/ Die Grünen	AfD	FDP	Die Linke	Sonstige
Gewichtete Basis	2.191	448	419	258	181	199	86	151
Ungewichtete Basis	2.191	473	372	270	204	198	88	137
**Ich habe volles Vertrauen in die Sicherheit von Impfungen.**	*Antworten in Prozent*							
Stimme überhaupt nicht zu	16	8	7	4	42	11	9	27
Stimme eher nicht zu	16	16	13	9	25	20	15	22
Stimme eher zu	37	39	41	51	20	39	39	32
Stimme voll und ganz zu	25	33	34	33	9	24	33	19
Weiß nicht / keine Angabe	5	4	5	4	4	7	4	1
Stimme überhaupt nicht / eher nicht zu	32	24	20	13	67	31	24	49
Stimme voll und ganz / eher zu	62	72	75	84	29	63	72	51

Anzahl Teilnehmer	Total	SPD	CDU/CSU	Bündnis 90/ Die Grünen	AfD	FDP	Die Linke	Sonstige
Gewichtete Basis	2.191	448	419	258	181	199	86	151
Ungewichtete Basis	2.191	473	372	270	204	198	88	137
**Impfungen sind effektiv für die Eindämmung von infektiösen Krankheiten.**	*Antworten in Prozent*							
Stimme überhaupt nicht zu	6	3	2	3	16	5	2	8
Stimme eher nicht zu	7	5	5	3	12	8	7	8
Stimme eher zu	35	36	31	31	40	41	39	30
Stimme voll und ganz zu	45	52	58	60	23	42	49	46
Weiß nicht / keine Angabe	6	4	3	4	9	4	2	7
Stimme überhaupt nicht / eher nicht zu	13	8	7	6	28	13	9	16
Stimme voll und ganz / eher zu	80	88	89	91	63	83	88	76

S1 Table. Survey Results: Vaccine readiness (in German)

Anzahl Teilnehmer	Total	SPD	CDU/CSU	Bündnis 90/ Die Grünen	AfD	FDP	Die Linke	Sonstige
Gewichtete Basis	2.191	448	419	258	181	199	86	151
Ungewichtete Basis	2.191	473	372	270	204	198	88	137
**Was Impfen anbelangt, vertraue ich darauf, dass staatliche Behörden immer im besten Interesse für die Allgemeinheit entscheiden.**	*Antworten in Prozent*							
Stimme überhaupt nicht zu	18	9	8	5	46	16	10	37
Stimme eher nicht zu	15	14	11	11	16	24	11	14
Stimme eher zu	36	42	38	46	22	36	43	27
Stimme voll und ganz zu	24	30	35	33	10	19	34	18
Weiß nicht / keine Angabe	7	4	8	5	6	6	3	5
Stimme überhaupt nicht / eher nicht zu	33	23	19	16	62	40	21	51
Stimme voll und ganz / eher zu	60	72	73	79	32	55	77	45

Anzahl Teilnehmer	Total	SPD	CDU/CSU	Bündnis 90/ Die Grünen	AfD	FDP	Die Linke	Sonstige
Gewichtete Basis	2.191	448	419	258	181	199	86	151
Ungewichtete Basis	2.191	473	372	270	204	198	88	137
**Wenn alle geimpft sind, brauche ich mich nicht auch noch impfen lassen.**	*Antworten in Prozent*							
Stimme überhaupt nicht zu	43	48	48	56	31	40	39	49
Stimme eher nicht zu	28	28	26	31	28	30	35	22
Stimme eher zu	11	10	12	4	12	14	12	7
Stimme voll und ganz zu	8	7	9	4	13	6	10	5
Weiß nicht / keine Angabe	11	7	7	4	16	10	5	17
Stimme überhaupt nicht / eher nicht zu	71	76	74	87	59	70	74	71
Stimme voll und ganz / eher zu	19	17	21	8	25	20	22	12

S1 Table. Survey Results: Vaccine readiness (in German)

Anzahl Teilnehmer	Total	SPD	CDU/CSU	Bündnis 90/ Die Grünen	AfD	FDP	Die Linke	Sonstige
Gewichtete Basis	2.191	448	419	258	181	199	86	151
Ungewichtete Basis	2.191	473	372	270	204	198	88	137
**Ich lasse mich impfen, weil ich damit auch Menschen mit einem schwachen Immunsystem schützen kann.**	*Antworten in Prozent*							
Stimme überhaupt nicht zu	13	6	8	6	32	13	4	21
Stimme eher nicht zu	11	9	11	4	19	14	6	10
Stimme eher zu	29	30	27	29	27	35	42	28
Stimme voll und ganz zu	41	51	52	59	16	32	41	37
Weiß nicht / keine Angabe	5	4	3	3	7	6	7	4
Stimme überhaupt nicht / eher nicht zu	24	15	19	10	51	27	10	31
Stimme voll und ganz / eher zu	70	81	79	88	43	67	83	65

Anzahl Teilnehmer	Total	SPD	CDU/CSU	Bündnis 90/ Die Grünen	AfD	FDP	Die Linke	Sonstige
Gewichtete Basis	2.191	448	419	258	181	199	86	151
Ungewichtete Basis	2.191	473	372	270	204	198	88	137
**Ob man sich impfen lässt oder nicht, ist eine rein persönliche Sache.**	*Antworten in Prozent*							
Stimme überhaupt nicht zu	6	7	8	11	2	4	4	7
Stimme eher nicht zu	20	25	22	32	10	18	27	14
Stimme eher zu	28	30	30	29	23	28	31	26
Stimme voll und ganz zu	41	35	35	24	60	46	34	48
Weiß nicht / keine Angabe	5	3	6	3	5	4	3	4
Stimme überhaupt nicht / eher nicht zu	26	32	30	43	12	22	31	21
Stimme voll und ganz / eher zu	69	65	65	53	83	74	65	74

S1 Table. Survey Results: Vaccine readiness (in German)

Anzahl Teilnehmer	Total	SPD	CDU/CSU	Bündnis 90/ Die Grünen	AfD	FDP	Die Linke	Sonstige
Gewichtete Basis	2.191	448	419	258	181	199	86	151
Ungewichtete Basis	2.191	473	372	270	204	198	88	137
**Impfen ist eine gemeinschaftliche Maßnahme, um die Verbreitung von Krankheiten zu verhindern.**	*Antworten in Prozent*							
Stimme überhaupt nicht zu	8	4	3	3	20	6	5	9
Stimme eher nicht zu	8	7	7	4	13	13	4	9
Stimme eher zu	33	33	31	29	34	38	38	32
Stimme voll und ganz zu	45	54	57	62	25	36	50	43
Weiß nicht / keine Angabe	5	2	3	3	9	7	3	6
Stimme überhaupt nicht / eher nicht zu	16	11	10	7	33	19	9	18
Stimme voll und ganz / eher zu	78	87	88	91	59	74	88	75

Anzahl Teilnehmer	Total	SPD	CDU/CSU	Bündnis 90/ Die Grünen	AfD	FDP	Die Linke	Sonstige
Gewichtete Basis	2.191	448	419	258	181	199	86	151
Ungewichtete Basis	2.191	473	372	270	204	198	88	137
**Viele pharmazeutische Unternehmen, Krankenhäuser und Mediziner:innen haben von der COVID-19 Pandemie übermäßig profitiert.**	*Antworten in Prozent*							
Stimme überhaupt nicht zu	5	5	7	7	2	4	3	5
Stimme eher nicht zu	15	21	15	23	5	15	15	13
Stimme eher zu	33	36	37	38	23	36	41	22
Stimme voll und ganz zu	36	28	33	19	65	33	31	44
Weiß nicht / keine Angabe	11	10	9	13	6	11	11	16
Stimme überhaupt nicht / eher nicht zu	20	26	22	30	7	19	18	18
Stimme voll und ganz / eher zu	69	64	70	57	88	69	72	66

S1 Table. Survey Results: Vaccine readiness (in German)

Anzahl Teilnehmer	Total	SPD	CDU/CSU	Bündnis 90/ Die Grünen	AfD	FDP	Die Linke	Sonstige
Gewichtete Basis	2.191	448	419	258	181	199	86	151
Ungewichtete Basis	2.191	473	372	270	204	198	88	137
**In Deutschland konnte die COVID-19 Pandemie durch Impfungen eingedämmt werden.**	*Antworten in Prozent*							
Stimme überhaupt nicht zu	13	5	7	3	37	13	3	23
Stimme eher nicht zu	13	10	10	6	20	17	11	16
Stimme eher zu	33	38	32	34	20	37	35	30
Stimme voll und ganz zu	33	38	45	51	12	24	41	23
Weiß nicht / keine Angabe	9	9	6	6	11	8	9	9
Stimme überhaupt nicht / eher nicht zu	26	15	17	9	57	30	14	39
Stimme voll und ganz / eher zu	66	76	77	85	32	61	76	53

Feldzeit: 01.03. - 05.03.2024
