# Supplementary material for: Vaccination readiness and political party preference in Germany: Trust, collective responsibility, and the populist radical right
Source: PLoS One. 2025 Jul 14;20(7):e0328045. doi: 10.1371/journal.pone.0328045 (PMC12258577; doi:10.1371/journal.pone.0328045)
Supplement: S2. Table — (PDF) [file pone.0328045.s002.pdf]

S2 Table. Survey Results: Vaccine readiness (in English)

Number of participants	Total	SPD	CDU/CSU	Bündnis 90/ Die Grünen	AfD	FDP	Die Linke	Sonstige
weighted	2.191	448	419	258	181	199	86	151
not weighted	2.191	473	372	270	204	198	88	137
**I have full confidence in the safety of vaccinations.**	*answers in percent*							
disagree completely	16	8	7	4	42	11	9	27
tend to disagree	16	16	13	9	25	20	15	22
tend to agree	37	39	41	51	20	39	39	32
agree completely	25	33	34	33	9	24	33	19
do not know / no response	5	4	5	4	4	7	4	1
disagree completely / tend to disagree	32	24	20	13	67	31	24	49
agree completely / tend to agree	62	72	75	84	29	63	72	51

Number of participants	Total	SPD	CDU/CSU	Bündnis 90/ Die Grünen	AfD	FDP	Die Linke	Sonstige
weighted	2.191	448	419	258	181	199	86	151
not weighted	2.191	473	372	270	204	198	88	137
**Vaccinations are effective for the containment of infectious diseases.**	*answers in percent*							
disagree completely	6	3	2	3	16	5	2	8
tend to disagree	7	5	5	3	12	8	7	8
tend to agree	35	36	31	31	40	41	39	30
agree completely	45	52	58	60	23	42	49	46
do not know / no response	6	4	3	4	9	4	2	7
disagree completely / tend to disagree	13	8	7	6	28	13	9	16
agree completely / tend to agree	80	88	89	91	63	83	88	76

S2 Table. Survey Results: Vaccine readiness (in English)

Number of participants	Total	SPD	CDU/CSU	Bündnis 90/ Die Grünen	AfD	FDP	Die Linke	Sonstige
weighted	2.191	448	419	258	181	199	86	151
not weighted	2.191	473	372	270	204	198	88	137
**When it comes to vaccinations, I always trust state authorities to decide in the best interest of the public.**	*answers in percent*							
disagree completely	18	9	8	5	46	16	10	37
tend to disagree	15	14	11	11	16	24	11	14
tend to agree	36	42	38	46	22	36	43	27
agree completely	24	30	35	33	10	19	34	18
do not know / no response	7	4	8	5	6	6	3	5
disagree completely / tend to disagree	33	23	19	16	62	40	21	51
agree completely / tend to agree	60	72	73	79	32	55	77	45

Number of participants	Total	SPD	CDU/CSU	Bündnis 90/ Die Grünen	AfD	FDP	Die Linke	Sonstige
weighted	2.191	448	419	258	181	199	86	151
not weighted	2.191	473	372	270	204	198	88	137
**If everyone is vaccinated, I don't need to get vaccinated.**	*answers in percent*							
disagree completely	43	48	48	56	31	40	39	49
tend to disagree	28	28	26	31	28	30	35	22
tend to agree	11	10	12	4	12	14	12	7
agree completely	8	7	9	4	13	6	10	5
do not know / no response	11	7	7	4	16	10	5	17
disagree completely / tend to disagree	71	76	74	87	59	70	74	71
agree completely / tend to agree	19	17	21	8	25	20	22	12

S2 Table. Survey Results: Vaccine readiness (in English)

Number of participants	Total	SPD	CDU/CSU	Bündnis 90/ Die Grünen	AfD	FDP	Die Linke	Sonstige
weighted	2.191	448	419	258	181	199	86	151
not weighted	2.191	473	372	270	204	198	88	137
**I get vaccinated because I can also protect people with a weak immune system.**	*answers in percent*							
disagree completely	13	6	8	6	32	13	4	21
tend to disagree	11	9	11	4	19	14	6	10
tend to agree	29	30	27	29	27	35	42	28
agree completely	41	51	52	59	16	32	41	37
do not know / no response	5	4	3	3	7	6	7	4
disagree completely / tend to disagree	24	15	19	10	51	27	10	31
agree completely / tend to agree	70	81	79	88	43	67	83	65

Number of participants	Total	SPD	CDU/CSU	Bündnis 90/ Die Grünen	AfD	FDP	Die Linke	Sonstige
weighted	2.191	448	419	258	181	199	86	151
not weighted	2.191	473	372	270	204	198	88	137
**Whether or not to get vaccinated is a purely personal decision.**	*answers in percent*							
disagree completely	6	7	8	11	2	4	4	7
tend to disagree	20	25	22	32	10	18	27	14
tend to agree	28	30	30	29	23	28	31	26
agree completely	41	35	35	24	60	46	34	48
do not know / no response	5	3	6	3	5	4	3	4
disagree completely / tend to disagree	26	32	30	43	12	22	31	21
agree completely / tend to agree	69	65	65	53	83	74	65	74

S2 Table. Survey Results: Vaccine readiness (in English)

Number of participants	Total	SPD	CDU/CSU	Bündnis 90/ Die Grünen	AfD	FDP	Die Linke	Sonstige
weighted	2.191	448	419	258	181	199	86	151
not weighted	2.191	473	372	270	204	198	88	137
**Vaccination is a community measure to prevent the spread of disease.**	*answers in percent*							
disagree completely	8	4	3	3	20	6	5	9
tend to disagree	8	7	7	4	13	13	4	9
tend to agree	33	33	31	29	34	38	38	32
agree completely	45	54	57	62	25	36	50	43
do not know / no response	5	2	3	3	9	7	3	6
disagree completely / tend to disagree	16	11	10	7	33	19	9	18
agree completely / tend to agree	78	87	88	91	59	74	88	75

Number of participants	Total	SPD	CDU/CSU	Bündnis 90/ Die Grünen	AfD	FDP	Die Linke	Sonstige
weighted	2.191	448	419	258	181	199	86	151
not weighted	2.191	473	372	270	204	198	88	137
**Many pharmaceutical companies, hospitals and medical doctors profited excessively from the COVID-19 pandemic.**	*answers in percent*							
disagree completely	5	5	7	7	2	4	3	5
tend to disagree	15	21	15	23	5	15	15	13
tend to agree	33	36	37	38	23	36	41	22
agree completely	36	28	33	19	65	33	31	44
do not know / no response	11	10	9	13	6	11	11	16
disagree completely / tend to disagree	20	26	22	30	7	19	18	18
agree completely / tend to agree	69	64	70	57	88	69	72	66

S2 Table. Survey Results: Vaccine readiness (in English)

Number of participants	Total	SPD	CDU/CSU	Bündnis 90/ Die Grünen	AfD	FDP	Die Linke	Sonstige
weighted	2.191	448	419	258	181	199	86	151
not weighted	2.191	473	372	270	204	198	88	137
**In Germany vaccinations were able to contain the COVID-19 pandemic.**	*answers in percent*							
disagree completely	13	5	7	3	37	13	3	23
tend to disagree	13	10	10	6	20	17	11	16
tend to agree	33	38	32	34	20	37	35	30
agree completely	33	38	45	51	12	24	41	23
do not know / no response	9	9	6	6	11	8	9	9
disagree completely / tend to disagree	26	15	17	9	57	30	14	39
agree completely / tend to agree	66	76	77	85	32	61	76	53

field time: 01.03. - 05.03.2024
