# Supplementary material for: Vaccination readiness and political party preference in Germany: Trust, collective responsibility, and the populist radical right
Source: PLoS One. 2025 Jul 14;20(7):e0328045. doi: 10.1371/journal.pone.0328045 (PMC12258577; doi:10.1371/journal.pone.0328045)
Supplement: S3. Table — (PDF) [file pone.0328045.s003.pdf]

S3 Table. COVID-19 Vaccinations received, listed by voting behavior in the Bundestag elections 2021

Number of participants	Total	SPD	CDU/CSU	Bündnis 90/ Die Grünen	AfD	FDP	Die Linke	Sonstige
weighted	2.171	443	415	258	180	198	83	151
not weighted	2.171	467	369	269	202	197	86	136
**How many times were you vaccinated against the COVID-19 virus, including "Booster"-vaccinations?**	*answers in percent*							
once	4	3	3	4	2	5	12	7
twice	17	15	16	12	12	19	23	18
three times	39	41	41	45	33	43	39	35
more than three times	24	33	34	32	12	24	21	17
not at all, due to health reasons	2	2	1	1	5	1	0	4
not at all, due to personal reasons	7	2	4	3	25	4	4	11
not at all, due to other reasons	3	2	0	1	7	2	1	4
I cannot remember	1	1	1	1	0	2	0	1
no response	2	1	1	0	2	0	0	2

field time: 01.03. - 05.03.2024
