# Supplementary material for: Vaccination readiness and political party preference in Germany: Trust, collective responsibility, and the populist radical right
Source: PLoS One. 2025 Jul 14;20(7):e0328045. doi: 10.1371/journal.pone.0328045 (PMC12258577; doi:10.1371/journal.pone.0328045)
Supplement: S4. Table — (PDF) [file pone.0328045.s004.pdf]

S4 Table. Number of ‘no responses’ listed by party voting behavior in the Bundestag elections 2021

Number of participants	Total	SPD	CDU/CSU	Bündnis 90/ Die Grünen	AfD	FDP	Die Linke	Sonstige
weighted	2.191	448	419	258	181	199	86	151
not weighted	2.191	473	372	270	204	198	88	137
**I have full confidence in the safety of vaccinations.**	*answers in percent*							
do not know / no response	5	4	5	4	4	7	4	1
**Vaccinations are effective for the containment of infectious diseases.**	*answers in percent*							
do not know / no response	6	4	3	4	9	4	2	7
**When it comes to vaccinations, I always trust state authorities to decide in the best interest of the public.**	*answers in percent*							
do not know / no response	7	4	8	5	6	6	3	5
**If everyone is vaccinated, I don't need to get vaccinated.**	*answers in percent*							
do not know / no response	11	7	7	4	16	10	5	17
**I get vaccinated because I can also protect people with a weak immune system.**	*answers in percent*							
do not know / no response	5	4	3	3	7	6	7	4

S4 Table. Number of 'no responses' listed by party voting behavior in the Bundestag elections 2021

Number of participants	Total	SPD	CDU/CSU	Bündnis 90/ Die Grünen	AfD	FDP	Die Linke	Sonstige
weighted	2.191	448	419	258	181	199	86	151
not weighted	2.191	473	372	270	204	198	88	137
**Whether or not to get vaccinated is a purely personal decision.**	*answers in percent*							
do not know / no response	5	3	6	3	5	4	3	4
**Vaccination is a community measure to prevent the spread of disease.**	*answers in percent*							
do not know / no response	5	2	3	3	9	7	3	6
**Many pharmaceutical companies, hospitals and medical doctors profited excessively from the COVID-19 pandemic.**	*answers in percent*							
do not know / no response	11	10	9	13	6	11	11	16
**In Germany vaccinations were able to contain the COVID-19 pandemic.**	*answers in percent*							
do not know / no response	9	9	6	6	11	8	9	9
field time: 01.03. - 05.03.2024								
