# Supplementary material for: Vaccination readiness and political party preference in Germany: Trust, collective responsibility, and the populist radical right
Source: PLoS One. 2025 Jul 14;20(7):e0328045. doi: 10.1371/journal.pone.0328045 (PMC12258577; doi:10.1371/journal.pone.0328045)
Supplement: S5. Table — with explication. Nine item averages according to political party. (DOCX) [file pone.0328045.s005.docx]

S5 Table and Explanation

**2) Political party averages of the nine items measuring vaccine readiness**

Table 2 presents the political party averages for each of the nine vaccine readiness measures. (A complete breakdown of the range of responses given by each group can be found in Supplements 1 and 2.) Viewed individually, each of these items reflects one aspect of vaccine readiness (either trust in vaccinations or collective responsibility). Results show that Green party voters expressed the most confidence in the safety of vaccinations (Q1), while AfD voters expressed the least confidence in this category. Trust in state authorities (Q3) was also lowest among AfD voters, and highest among Green party voters, with only marginal differences from other mainstream parties (SPD, CDU/CSU) and from The Left party.

Little to no hesitancy was expressed by any of the groups on three of the measures (Q2 effectiveness of vaccines, Q4 rejecting free-riding, and Q7 understanding vaccination as a community project). With respect to the effectiveness of vaccines, CDU/CSU and Green party voters expressed the most confidence, and these voters were also the least likely to dismiss the need to get vaccinated even if all others were vaccinated. Green party voters were the most likely to see vaccination as a community measure (3.55), followed closely by the CDU/CSU (3.44), SPD (3.39) and The Left party (3.38). In each of these areas, AfD voters expressed views that reflected vaccine readiness, but they did so to a lesser degree (2.70) than other voters. FDP voters had the second lowest vaccine readiness score on all measures except for Q4; however, these voters expressed fundamentally positive views in all but two of the measures (Q6 and Q8), where considerable hesitancy was expressed across all parties. Participants from all parties tended to agree that the decision to get vaccinated is a personal decision, and this disagreement was expressed most emphatically by AfD (reverse-coded: 1.52) and FDP voters (reverse-coded:1.80). Average scores from all parties also showed considerable agreement with the notion that pharmaceutical companies, hospitals, and medical doctors profited excessively from the COVID-19 pandemic. Here, too, AfD and FDP voters agreed the most emphatically, signaling a lower level of vaccine readiness (reverse-coded: AfD=1.41; FDP=1.90), while Green voters proved to be the least suspicious of unjustified profits (reverse-coded: 2.21).

Group averages indicate a general acknowledgment that the protection of those with a weak immune system is a reason to get vaccinated (Q5), though AfD voters were the least likely to acknowledge this (2.30). AfD voters also stood out in their rejection (2.09 average) of the notion that vaccinations served to contain the spread of the COVID-19 in Germany (Q9). Here responses were generally positive (2.79 or higher) with the exception of AfD voters, whose tendency to agree was considerably lower (2.09).

Notes:

Averages are rounded to the second decimal place, rounded up from 0.005.

***These scores represent the unadjusted averages before the application of additional controls.**

** Indicates that the items were reverse coded so that a high response signifies a positive result (e.g. high community orientation and high trust in pharmaceutical and medical professional and institutions).

**Table 2. Vaccine readiness: political party averages of each separate item***

| **Please indicate the extent to which you agree or disagree with the following statements:** | **AfD** | **CDU/CSU** | **FDP** | **Green** | **Left** | **SPD** |
| --- | --- | --- | --- | --- | --- | --- |
| 1. I have full confidence in the safety of vaccinations. | 1.96 | 3.07 | 2.81 | 3.17 | 3.00 | 3.01 |
| 2. Vaccinations are effective for the containment of infectious diseases. | 2.77 | 3.51 | 3.26 | 3.54 | 3.39 | 3.42 |
| 3. When it comes to vaccinations, I always trust state authorities to decide in the best interest of the public. | 1.97 | 3.09 | 2.61 | 3.13 | 3.04 | 2.98 |
| 4. If everyone is vaccinated, I don't need to get vaccinated.** | 2.93 | 3.20 | 3.15 | 3.46 | 3.07 | 3.26 |
| 5. I get vaccinated because I can also protect people with a weak immune system. | 2.30 | 3.27 | 2.92 | 3.44 | 3.29 | 3.31 |
| 6. Whether or not to get vaccinated is a purely personal decision.** | 1.52 | 2.03 | 1.80 | 2.31 | 2.01 | 2.05 |
| 7. Vaccination is a community measure to prevent the spread of disease. | 2.70 | 3.44 | 3.12 | 3.55 | 3.38 | 3.39 |
| 8. Many pharmaceutical companies, hospitals and medical doctors profited excessively from the COVID-19 pandemic.** | 1.41 | 1.95 | 1.90 | 2.21 | 1.89 | 2.03 |
| 9. In Germany vaccinations were able to contain the COVID-19 pandemic. | 2.09 | 3.24 | 2.79 | 3.41 | 3.27 | 3.21 |
